# Assessment of fidelity in individual level behaviour change interventions promoting physical activity among adults: a systematic review

**DOI:** 10.1186/s12889-017-4778-6

**Published:** 2017-10-02

**Authors:** Jeffrey D. Lambert, Colin J. Greaves, Paul Farrand, Rosina Cross, Anne M. Haase, Adrian H. Taylor

**Affiliations:** 1 University of Exeter Medical School, St Luke’s Campus, Magdalen Road, Exeter, EX1 2LU UK; 2 Clinical Education, Development and Research (CEDAR); Psychology, University of Exeter, Exeter, EX4 4QG UK; 3 Department for Health, University of Bath, Wessex House 6.9, Claverton, Bath, BA2 7AY UK; 4 Centre for Exercise, Nutrition and Health Sciences, University of Bristol, 8 Priory Road, Bristol, BS8 1TZ UK; 5 Plymouth University, N6, ITTC, Tamar Science Park, Plymouth, Devon PL6 8BX UK

**Keywords:** Fidelity, Physical activity, Adults, Behaviour change, Systematic review, Behavioural intervention

## Abstract

**Background:**

Behaviour change interventions that promote physical activity have major implications for health and well-being. Measuring intervention fidelity is crucial in determining the extent to which an intervention is delivered as intended, therefore increasing scientific confidence about effectiveness. However, we lack a clear overview of how well intervention fidelity is typically assessed in physical activity trials.

**Methods:**

A systematic literature search was conducted to identify peer - reviewed physical activity promotion trials that explicitly measured intervention fidelity. Methods used to assess intervention fidelity were categorised, narratively synthesised and critiqued using assessment criteria from NIH Behaviour Change Consortium (BCC) Treatment Fidelity Framework (design, training, delivery, receipt and enactment).

**Results:**

Twenty eight articles reporting of twenty one studies used a wide variety of approaches to measure intervention fidelity. Delivery was the most common domain of intervention fidelity measured. Approaches used to measure fidelity across all domains varied from researcher coding of observational data (using checklists or scales) to participant self-report measures. There was considerable heterogeneity of methodological approaches to data collection with respect to instruments used, attention to psychometric properties, rater-selection, observational method and sampling strategies.

**Conclusions:**

In the field of physical activity interventions, fidelity measurement is highly heterogeneous both conceptually and methodologically. Clearer articulation of the core domains of intervention fidelity, along with appropriate measurement approaches for each domain are needed to improve the methodological quality of fidelity assessment in physical activity interventions. Recommendations are provided on how this situation can be improved.

**Electronic supplementary material:**

The online version of this article (10.1186/s12889-017-4778-6) contains supplementary material, which is available to authorized users.

## Background

Lack of physical activity is a key risk factor for mortality, associated with approximately 5.3 million deaths per year worldwide [1]. Regular maintenance of physical activity can also have significant implications for physical and mental health including reduced risk of depression [2] reduced risk of cardiovascular disease, and weight loss [3] and physical activity promotion is considered to be the “best buy” for public health [4]. In the UK, guidance from the Chief Medical Officer currently recommends at least 150 min of moderate to vigorous physical activity (MVPA) per week. Despite this, national levels of MVPA in the UK are low with only 39% of men and 29% of women achieving this target [5].

Individual level (one to one and group-based) behavioural interventions are a key strategy for increasing physical activity, however, there is considerable variation in their reported effectiveness [6–8]. This may be due to the fact that behavioural interventions for physical activity are often complex (with many interacting factors), and are therefore challenging to design and implement [9]. These interacting factors moderate and mediate study outcomes, and include theoretical mechanisms (e.g. motivation or confidence) and contextual factors (e.g. participant demographics) [10]. Another, key moderator of study outcomes is intervention fidelity - the extent to which a behavioural intervention was designed, implemented and received as intended [11, 12].

Inadequate attention to the assessment of intervention fidelity can increase the risk of type 1 and type 2 errors and result in spurious conclusions about intervention effectiveness [11]. As well as allowing more accurate judgements about effectiveness [13], assessing fidelity can also facilitate easier replication and implementation of behavioural interventions in real world settings [9]. The UK, Medical Research Council (MRC) guidelines emphasise the importance of fidelity assessment when interpreting outcomes [14, 15]. One framework specifically developed for individual level behaviour change interventions was developed by the NIH Behaviour Change Consortium (BCC). The BCC conceptualised fidelity across five core domains: Study Design, Provider Training, Intervention Delivery, Intervention Receipt and Enactment. Study Design is concerned with whether a study adequately tests its hypotheses in relation to its underlying theoretical and clinical processes. Provider Training involves standardizing training between providers and ensuring they are trained to clear criteria and monitored over time. Intervention Delivery involves assessing and monitoring differentiation (differences between the intervention and any comparison treatments), competency (skills set of provider), and adherence (delivery of intended components). Intervention Receipt refers to whether the intervention was understood and ‘received’ by participants and enactment refers to whether participants used intervention related skills in day to day settings [11, 13, 16]. The NIH BCC framework provides guidance for the assessment, enhancement and monitoring of fidelity, however, the focus of the present review is on assessment. Focussing on assessment is important to ensure proposed strategies to enhance fidelity (e.g. those recommended by the NIH BCC) have indeed been successful (e.g. did the provision of a treatment manual result in adequate adherence to treatment components?) and also facilitates accurate monitoring of fidelity over time.

If the assessment of intervention fidelity is important, then agreement on what constitutes fidelity in physical activity interventions is clearly needed. In addition, recommendations for good practice could help to reduce the risk of bias when assessing fidelity [17, 18]. Despite this, reviews investigating fidelity assessment in health behaviour research [16, 19] self-management [20, 21], mental health [22] school based drug abuse prevention [23] and physical activity [24, 25] have revealed that there is considerable variability in the conceptualisation and measurement of intervention fidelity. For example, in a review of diabetes self-management interventions, it was reported that intervention fidelity was assessed inconsistently, using a range of different concepts, including adherence to intervention content, duration, coverage and quality of programme delivery. There was also heterogeneity in measurement, with a variety of approaches such as participant self-report, researcher observation, and provider self-report [20].

A variety of ways to conceptualise [11, 13, 16, 26] and measure [22, 27] fidelity in behavioural interventions have been suggested. Previous studies have also reviewed the theoretical basis of physical activity counselling interventions, and competency level of the interventionists [24] and highlighted the importance of assessing fidelity in physical activity interventions based on motivational theories [25]. However, to the best of our knowledge, a review of whether and how fidelity has been assessed (using the NIH BCC framework) in physical activity interventions, along with an appraisal of the quality of these approaches and association to outcomes is lacking. An overview of this field would provide intervention developers with a foundation to improve fidelity assessment of their own interventions, and provide researchers and reviewers with a means to assess the extent to which reported intervention processes are a) delivered and b) responsible for study outcomes.

The current review has four key aims. Firstly, to identify and summarise (using the NIH BCC framework) how behavioural interventions to promote physical activity in adults have conceptualised and measured fidelity. Second, to summarise the reported results of fidelity assessments. Third to summarise any reported associations of fidelity and other intervention outcomes. Fourth, to critically appraise the methodological approaches identified.

## Methods

### Searches

A search of the databases PsychInfo, PsychArticles, MEDLINE, Embase, and Google Scholar was undertaken in March 2017 for all studies published in English up to that date. Searches were carried out on titles, abstracts and keywords using proximity and wildcard operators to maximise the range of potential studies. Search terms for intervention fidelity (Additional file 1) were informed by previous reviews [16, 20, 28] and consisted of synonyms for intervention fidelity (e.g. treatment integrity) combined with those for ‘exercise’ (e.g. physical activity). Additional searches were carried out by citation searching of included papers.

### Study inclusion/exclusion criteria

Retrieved studies were included based on the following criteria: [1] Mentioning fidelity (or related term) in the title, abstract or keywords either as a main focus of the study or as a nested study (e.g. as an analysis conducted within a trial or feasibility study); [2] Individual level behavioural interventions [29] designed to increase any type of physical activity; [3] Study focussed only on physical activity and no other behaviours (e.g. diet, smoking); [4] Study involving adults aged 18 or over; [5] Peer reviewed publications in English published up to March 2017 (no time frame imposed) [6] RCTs, observational studies pre-post studies, case-controlled or other quasi-experimental studies. Comparison groups could include usual care, no intervention or other interventions as the present study was only interested in the main intervention group. Studies were excluded if the intervention consisted of structured exercise alone or behavioural support plus structured physical activity (e.g. exercise classes) (Additional file 2).

### Study selection

All titles and abstracts were screened by the lead author (JL) with 10% independently screened by another co-author (RC). All full texts were also screened independently by JL and RC. Inter-rater reliability was calculated using the AC1 statistic [30] and disagreements were resolved by discussion and, if necessary, mediated by a third author (CG).

### Data extraction and synthesis

Data extraction and synthesis was guided by previous recommendations on the conduct of narrative synthesis in systematic reviews. Narrative synthesis is an approach used in systematic reviews to textually summarise findings from the included studies [31]. Characteristics of the main intervention study (i.e. study design, population, intervention, and physical activity outcome) were extracted in addition to fidelity data. Borrelli (2011) provides the latest iteration of the NIH BCC treatment fidelity checklist [11, 13, 16] (now referred to as the ‘treatment fidelity assessment and implementation plan’). Items pertaining only to fidelity assessment (within the domains of design, training, delivery, receipt and enactment) were used to organise and summarise the descriptions of author attempts at assessing fidelity, methods used to collect data and fidelity outcomes (Table 1). All items from the treatment fidelity assessment and implementation plan were not used as they referred to aspects not related to assessment (e.g. use of a treatment manual). Although important, these items relate to ‘enhancing’ as opposed to ‘assessing’ fidelity. Nvivo (version11) was used to organise the data.

**Table 1:** Fidelity Assessment and Quality Appraisal Criteria

NIH BCC Domain	Assessment Method^a^	Quality criteria^b^
Treatment Design	Assess whether intervention protocol/manuals reflect the underlying theoretical model or clinical guidelines	Prior to study implementation, investigators, and optimally a protocol review group or panel of experts, should review their protocols or treatment manuals to ensure that the active ingredients of the intervention are fully operationalized
		The degree to which the measures reflect the hypothesized theoretical constructs and mechanisms of action should be assessed
Training providers	Assess provider skills acquisition	Ensure providers are trained to a well-defined, a priori performance criterion. Provider role-plays with standardized patients should be evaluated for both adherence to treatment components and adherence to process (e.g., interactional style)
	Assess and monitor provider skills maintenance	
Delivery of treatment	Assess if provider adhered to intervention plan, or in the case of computer delivered intervention, method to assess participants contact with information	Adherence to treatment components and competence to deliver the treatment in the manner specified
	Assess non-specific treatment effects	Direct observation evaluated according to criteria developed a priori
	Assess whether or not the active ingredients were delivered	Raters of the audiotapes or videotapes should be skilled in treatment delivery as well as in more subtle aspects of the intervention and the treatment manual.
	Assess whether or not proscribed components were delivered (e.g. components that were unnecessary or unhelpful)	Raters of the audiotapes or videotapes should be independent of the study
		Raters of the audiotapes or videotapes should be blind to treatment assignment, participant progress and outcomes, and provider identity.
		Interrater reliability of raters of the audiotapes or videotapes should be conducted
Receipt of Treatment	Assess degree to which participants understood intervention	Assessment of treatment receipt involves verifying the participants’ understanding of the information provided in the treatment and verifying that they can use the skills and recommendations discussed. This could include written verification (pre–post-tests), using audio visuals (repeat information orally and visually), and behavioural strategies (role-plays skills with feedback).
	Assess participants ability to perform the intervention skills	
Enactment of treatment skills	Assess participant performance of intervention skills in setting in which the intervention is applied	Objective observation to determine if participants were using behaviour change techniques in relevant day to day settings
All		Psychometric properties

^a^Only items relating to fidelity assessment were taken from the Treatment fidelity assessment and implementation plan, ^b^Quality criteria informed by general recommendations made by [11, 13, 16, 17, 22]

As no specific criteria for appraising the quality of fidelity assessment exist, studies were critically appraised based on criteria suggested in previous studies to represent good practice when assessing fidelity [11, 13, 16, 17, 22]. The more general recommendations made in the NIH BCC papers were used to create a checklist by the study authors. This aimed to provide a sense of the ‘quality’ of the application of each method. The checklist items assessed the presence or absence of good practice methods for each study and confirmed how robust the fidelity measures were (Table 1). There were eleven criteria overall, with two for design, one for training, six for delivery, one for receipt, and one for enactment and one applied to all domains.. A previous checklist has been developed (i.e. the treatment fidelity assessment and implementation plan) to quantify the extent to which studies have assessed monitored and enhanced fidelity according to the five domains of the BCC framework [11, 13, 16]. However, this checklist was not appropriate for the purpose of appraising the quality of the assessment measured, as they did not include more specific items relating to the methodological quality of the fidelity measures themselves (e.g. method used to collect fidelity data). Data from all included studies was independently extracted and appraised by two authors and compared. Discrepancies were resolved by discussion with a third author.

## Results

### Included studies

**Fig. 1:**
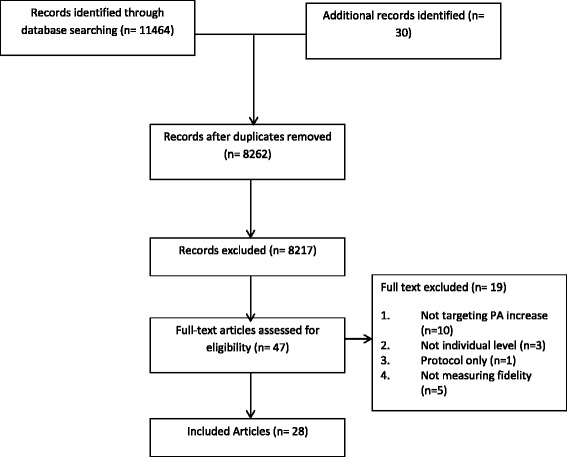
PRISMA Flow chart of selection process for review [64]

As highlighted in the PRISMA flow chart (Fig. 1), the search identified 11,464 records. Once duplications were removed, 8262 records remained. After title and abstract screening, 47 full texts were examined further with 28 articles describing 21 physical activity interventions included in the review. Inter-rater reliability for titles and abstracts was excellent (AC1 = 0.99) but poor for full text (AC1 = 0.23). However further discussion revealed a systematic difference in the way that one inclusion criterion was being applied (focussing on physical activity as an outcome rather than as a focus of the intervention). After clarification, the full text screening yielded perfect agreement (AC1 = 1).

### Study characteristics

16 studies were RCT’s [32–48] and five were one arm (pre-post) trials [49–53]. All studies reported some form of behavioural support which aimed to increase the uptake of physical activity (e.g. information about health benefits, goal setting, self-monitoring, and pedometers). Studies used motivational interviewing (MI) [34, 37, 39, 48, 52, 53], Cognitive Behaviour Therapy (CBT) [39], and guided self-management based on a range of theoretical approaches including social cognitive theory (SCT) [36, 44, 48, 50, 51], theory of planned behaviour (TPB) [47, 51], self-determination theory (SDT) [37, 41, 43], the trans-theoretical model (TTM) [36, 39, 40, 42, 47] and self-regulation theory [38, 46]. Physical activity was assessed using self-report [32–34, 36, 37, 39–41, 44, 47, 48, 50, 52], or objective measures of physical activity (e.g. pedometers or accelerometers, pedometers or heart rate monitors (36,39,47,49). Of the studies that reported outcomes relating to physical activity, nine reported an increase [32, 34–36, 38, 41, 47, 48, 50], and three reported no increase in physical activity [37, 40, 46]. Studies included participants with a range of ages (36 to 81) and consisted of inactive employees [32], university staff [40] inactive post-partum women [33], African American women [48] patients recovering from spinal cord injury [50], people with co morbid depression and multiple sclerosis [34], people aged over 50 [36, 39, 42], people from deprived communities [37], people with type 2 diabetes [38, 51], people with intellectual disabilities [49], primary care patients [46, 52]) overweight adults [35], inactive adults [41, 44, 47], people with heart failure [53] and people Huntington’s disease [43]. The mode of intervention delivery included face to face [32, 34, 36–38, 42–44, 46, 48–53], online [33, 35, 40, 44, 47, 51], email [32, 40] post [41] and by telephone [33, 34, 36, 39, 48, 53], with both individual (one to one) and group based intervention formats. A more detailed description of each included study can be found in Additional file 3.

### Measurement of intervention Fidelity

Overall, 66 approaches to measuring fidelity were identified across the 21 studies with 52 approaches measuring delivery fidelity, eight measuring enactment, four measuring receipt, two measuring training fidelity and no approaches assessing design fidelity. Table 2 provides an overview of the fidelity measures identified. It is important to note that many studies contained multiple concepts or measurement approaches.

**Table 2:** Summary of Intervention Fidelity Measures

NIH BCC Domain (n)	Assessment Criteria (n)	What measured (n)	How Measured (n)	Study^a^
Treatment Design (0)	Assess whether intervention protocol/manuals reflect the underlying theoretical model or clinical guidelines (0)	N/A	N/A	N/A
Training providers (2)	Assess provider skills acquisition (2)	Provider confidence to deliver intervention (1)	Provider self-report (1)	[46]
		Provider competence to deliver intervention (1)	Assessment of provider (1)	[42]
	Assess and monitor provider skills maintenance (0)	N/A	N/A	
Delivery of treatment (51)	Assess if provider adhered to intervention plan, or in the case of computer delivered intervention, method to assess participants contact with information (28)	Number of email messages delivered (1)	Researcher observation (1)	[32]
		Number of email messages/intervention materials read/received (3)	Participant self-report (3)	[32, 40, 41]
		Number of website log ins (4)	Automatically tracked (4)	[33, 35, 40, 54]
		Number of automated calls (1)	Automatically tracked (1)	[48]
		Time spent on website (2)	Automatically tracked (2)	[40, 47]
		Number of pages viewed (2)	Automatically tracked (2)	[33, 40]
		Number of website modules read (1)	Participant self-report (1)	[40]
		Provider rating of satisfaction with delivery (2)	Provider self-report (2)	[50, 52]
		Number of intervention sessions delivered (5)	Audio observation (3)	[33, 34, 53, 65]
			Researcher observation (1)	[48]
			Provider self-report (1)	[48]
		Time of intervention sessions delivered (6)	Audio observation (3)	[33, 65], [34] [53]
			Provider self-report (3)	[52] [36] [43]
		Percentage of intervention script adhered to (1)	Researcher observation (1)	[49]
	Assess non-specific treatment effects (6)	Rating of provider spirit/competence (4)	Audio observation (4)	[34, 37, 48, 53]
		Participant rating of provider support (2)	Provider self-report (2)	[36, 39]
	Assess whether or not the active ingredients were delivered (15)	Rating of intervention components delivered (2)	Audio observation (1)	[43]
			Provider self-report (1)	[43]
		Checklist of intervention components delivered (7)	Researcher observation (2)	[33, 51]
			Audio observation (1)	[48]
			Provider self-report (4)	[38, 42, 43, 52],
		Number of intervention components delivered (6)	Audio observation (5)	[34, 37, 38, 48, 53]
			Provider self-report (1)	[36]
	Assess whether or not proscribed components were delivered (e.g. components that were unnecessary or unhelpful) (3)	Number of proscribed intervention components delivered (3)	Audio observation (3)	[34, 37, 48]
Receipt of Treatment (4)	Assess degree to which participants understood intervention (1)	Participant perceived understanding of intervention skills (1)	Participant self-report (1)	[50]
	Assess participants ability to perform the intervention skills (3)	Participant demonstration of knowledge or skills acquired (1)	Researcher observation (1)	[49]
		Participant perceived efficacy to perform intervention skills (2)	Participant self-report (2)	[38, 50]
Enactment of treatment skills (8)	Assess participant performance of intervention skills in setting in which the intervention is applied (8)	Number of participants using intervention materials (e.g. log books, worksheets, pedometers) (3)	Participant Self report (2)	[32, 41]
			Automatically tracked (1)	[48]
		Number of times intervention materials used (e.g. log books, worksheets, pedometers, online self-monitoring) (2)	Automatically tracked (2)	[35, 53]
		Checklist of participant use of specified intervention techniques (e.g. action planning, self-monitoring) (2)	Participant Self report (2)	[35, 38]
		Participant rating of agreement with using intervention techniques (1)	Participant Self report (1)	[50]

^a^Some studies contain multiple measurement approaches

#### Design and training

No studies reported assessing design fidelity (the extent to which the intervention content reflected the underlying theory or logic model) and only two studies reported assessing training fidelity (the level of provider competence to deliver the intended intervention content before delivery). Training fidelity was assessed in one study by measuring provider competence using a 20 point checklist to assess whether or not providers adhered to the intervention protocol during practice sessions [46]. In the other study, it was measured using an eight item self-report scale to assess perceived provider confidence to deliver the intended intervention content [42]. One study reported an increase in provider confidence (training fidelity) in delivery of the intervention [42] the other study did not report the fidelity outcome, but stated that a minimum level of competence was required before delivering the intervention [46].

#### Delivery (human provider)

20 studies measured delivery of human providers. These included using self-report by providers to measure the presence or absence [38, 42, 43, 52], frequency [36], or delivery quality [43] of intervention components, or using observation by researchers to assess the presence or absence [33, 48, 51], frequency, [34, 37, 38, 48, 53] or delivery quality [43] of intervention components. Two studies reported measuring the provider’s satisfaction with his or her own intervention delivery [50, 52] and two studies reported measuring the participants’ satisfaction with intervention delivery [36, 39]. Four studies reported measuring researcher observed rating of provider competence/spirit (i.e. the interpersonal skills of the provider) [34, 37, 48, 53] and three studies reported using researcher assessment of the number of proscribed behaviours used (e.g. arguing/giving advice without permission) [34, 37, 48]. Seven studies assessed the treatment dose delivered (i.e. length of time or number of sessions) [33, 34, 36, 43, 48, 52, 53]. One study assessed provider adherence to an intervention script, although it was not clear how this was measured [49]. Data relating to delivery was obtained using provider [37, 38, 42, 43, 50, 52], and participant self-report [32, 36, 39–41, 44], as well as audio recordings [34, 37, 38, 43, 48, 53], video recordings [51], and direct observations [33, 49] by researchers. Provider interviews [52] were also used. Approaches to sampling varied with some studies taking a sample of sessions from the trial population [34, 37, 38, 48] and others sampling the whole trial population [32, 35, 36, 38, 50, 52]. All studies using observational methods opted to apply coding procedures to a selected sample of recordings (or transcripts), rather than using data from all possible intervention sessions. Overall, of the studies reporting fidelity outcomes, many reported adequate intervention delivery [32, 34–37, 52]. One study found adequate levels of fidelity for checklist of intervention components (>70%) and rating of competence measured by researchers. For the same study, less than adequate fidelity was found for an MI intervention measured by trained researchers using a ratio of number of ‘adherent’ vs ‘not-adherent’ intervention components [48]. Two studies contrasted delivery assessments made using different methods. One reported low levels of delivery fidelity using objective rating of audio transcripts (with 44% of intervention components delivered as intended), but with provider self-reports indicating high delivery fidelity (100% of intervention components delivered as intended) [38]. Another study found that provider self-assessment scores were higher than those assigned by an independent rater [43].

#### Delivery (web-based)

For web-based interventions, automatically tracked website log ins [33, 35, 40, 54], time spent on the website [40, 47] automated telephone calls sent [48] or number of pages viewed [33, 40] were recorded electronically, with one study reporting ‘modules used’ with participant self-report [40]. Three studies also measured self-reported receipt of emails [32, 40, 41].

#### Receipt

There were three approaches to assessing intervention receipt, these included; participant demonstration of knowledge or skills acquired [49], perceived understanding of intervention content [50], and participant confidence (self-efficacy) to perform skills taught by the intervention [38, 50]. Approaches to sampling for assessment of intervention receipt included sampling the whole trial population at the end of each session and ten days later [49] and assessing the whole trial population at the end of the trial [38, 50]. One study reported a small increase (from 86.2% to 89.4%) in perceived ability to carry out intervention skills [50]. Another study found that 73.5 to 100% of participants achieved the learning objectives of any given session [49].

#### Enactment

Enactment measures included participant self-reports or automatic tracking of using the intervention materials (e.g. log books, worksheets, pedometers) [32, 41, 48, 53] or of using specified intervention techniques (e.g. action planning/self-monitoring) [35, 38, 50]. One study also measured participant use of self-monitoring using electronically recorded data from the intervention website [35]. All studies that measured enactment collected data from the whole trial population. Studies that assessed enactment reported a range from 35.3% to 60% [32, 41] of participants regularly using intended intervention techniques post intervention. One study found a non-significant increase in the self-reported use of action planning techniques from pre to post intervention (4.6 to 6.8 on a 9 point scale) [50] and one study reported that all participants used intervention techniques as intended at 6 and 12 months follow up [38]. Finally, another study reported 71.9% of participants using intervention materials (accelerometers) [49].

### Intervention Fidelity in relation to physical activity (and other study outcomes)

#### Delivery in relation to physical activity

Only three studies assessed the relationship between delivery fidelity and physical activity. One study found a positive association between MI fidelity (counts of adherent (and non-adherent) components of intervention components and spirit of delivery) with objectively measured total energy expenditure (TEE) (*p* = 0.027) [37]. Two studies found no significant relationship between the number of intervention components delivered (based on coding of audio observations) and levels of self-reported and objective physical activity [38, 48].

#### Delivery in relation to other outcomes

Only one study looked at the relationship between number of intervention components delivered and participant confidence in using intervention strategies, intention to be physically active and affective attitude towards physical activity [38].

### Critical appraisal of intervention Fidelity measurement practices

A summary of critical appraisal criteria for each dimension of the BCC framework, and the number of studies meeting the criteria can be seen in Table 3.

**Table 3:** Critical appraisal of fidelity measures

BCC (number of studies identified)	Criteria (Bellg et al., 2004; Borrelli, 2011b; Borrelli et al., 2005)	Number of studies meeting criteria	
Design (0)	Prior to study implementation, investigators, and optimally a protocol review group or panel of experts, should review their protocols or treatment manuals to ensure that the active ingredients of the intervention are fully operationalized.	N/A	
	The degree to which the measures reflect the hypothesized theoretical constructs and mechanisms of action should be assessed.	N/A	
Training (2)	Ensure providers are trained to a well-defined, a priori performance criterion. Provider role-plays with standardized patients should be evaluated for both adherence to treatment components and adherence to process (e.g., interactional style).	1	[46]
Delivery (20)	Adherence to treatment components and competence to deliver the treatment in the manner specified	4	[34, 37, 48, 53].
	Direct observation evaluated according to criteria developed a priori	8	[33, 34, 37, 38, 43, 48, 49, 51]
	Raters of the audiotapes or videotapes should be skilled in treatment delivery as well as in more subtle aspects of the intervention and the treatment manual.	6	[34, 36, 37, 48, 51, 53]
	Raters of the audiotapes or videotapes should be independent of the study	3	[37, 38, 53]
	Raters of the audiotapes or videotapes should be blind to treatment assignment, participant progress and outcomes, and provider identity.	0	
	Interrater reliability of raters of the audiotapes or videotapes should be conducted	3	[38, 43, 51]
Receipt (3)	Assessment of treatment receipt involves verifying the participants’ understanding of the information provided in the treatment and verifying that they can use the skills and recommendations discussed. This could include written verification (pre–post-tests), using audio visuals (repeat information orally and visually), and behavioural strategies (role-plays skills with feedback).	1	[49]
Enactment (7)	Objective observation to determine if participants were using behaviour change techniques in relevant day to day settings	1	[35]
All [21]	Psychometric properties	6	[34, 36, 37, 48, 50, 53]

No studies assessed design fidelity, so by default none met these criteria.

Of the two studies that assessed training, only one met this criterion [46] by getting providers to do a practice role play and marking it against a checklist of intervention techniques. Of the 20 studies that assessed delivery fidelity, only four measured adherence to treatment components and competence to deliver the treatment in the manner specified [34, 37, 48, 53]. This was done using the Motivational Interviewing Treatment Integrity Scale (MITI) which measures both usage of MI techniques and ‘spirit’ of delivery (i.e. use of a person-led, empathy-building interactive style). This is important to ensure effects are due to treatment rather than to different interactional styles [13]. Some form of direct observation to evaluate delivery against a priori criteria (e.g. using audio/video tapes) was reported in eight studies [33, 34, 37, 38, 43, 48, 49, 51], enhancing the reliability of the data. Of the eight studies that used direct observation, credible data supporting the competence of the raters used to assess delivery fidelity (e.g. previous training in the intervention) was evident in six. Such evidence included mentions of having expertise in health behaviour change [51], being trained in MI [34, 37, 48] and being the ‘intervention director’ [36, 53]. Rater independence was only present in three studies [37, 38, 53] where external raters who were not otherwise involved in the study were employed to provide a more objective rating of fidelity. Evidence of rater blinding from providers, participants or outcomes was not reported in any of the studies and interrater reliability of raters was only reported for three studies, one reporting a Cohens Kappa of 0.60 [51] and the other two reporting percentage agreement scores ranging from 75% to 100% agreement [38, 43]. No studies met all six of these criteria and only two studies met at least four out of the six (11%). Of the three studies which measured receipt, only one study made use of knowledge tests by providing multiple choice tests as well as free text questions [49]. Finally, for the seven studies which measured enactment, only one study met the criterion of ‘objective observation to determine if participants were using behaviour change techniques in relevant day to day settings’ by counting the amount of times steps were logged by participants on a website [35]. Across all 21 identified studies, the psychometric properties of instruments used to measure any type of intervention fidelity were only reported for six studies. This involved either reporting internal consistencies [36, 50], intraclass correlations [48] or referencing the use of previously validated and reliable scales [34, 37, 48, 53].

## Discussion

### Summary of findings

This review systematically identified and summarised the range of concepts and methods used to assess intervention fidelity in interventions to increase physical activity and critically appraised the methods used. Only twenty eight articles reporting twenty one studies were identified which had explicitly examined intervention fidelity, suggesting an overall lack of attention to this issue in the field.

A range of different ways to assess intervention fidelity were identified, with delivery of intervention components being the most frequent. The concepts measured often deviated from those identified by the BCC Framework [13]. For example, there was a lack of clear distinction between fidelity of training and fidelity of delivery and no studies assessed every aspect of fidelity.

A wide range of approaches were used to measure fidelity, with data collection measures ranging from researcher coding of observational data (using checklists or scales) to participant self-report measures, to simple counting of sessions attended. A mixture of provider self-report and audio observation were most common for delivery and participant self-report was most common for receipt and enactment. However, there was an overall lack of methodological rigour in the approaches used for data collection (e.g. lack of attention to psychometrics and use of untrained, potentially biased raters) when appraised against a priori quality criteria for fidelity assessment.

### Relation to other literature/interpretations

The lack of attention, consistency and rigour in the conceptualisation and measurement of fidelity in physical activity interventions found in this review confirms previous findings. For example, a recent scoping review found that only 5% of published articles addressed the issue of fidelity in motivational physical activity interventions [25]. This also resembles findings in other behavioural domains [18, 20, 22]. For instance, in a review of fidelity in diabetes self-management interventions, only fifteen studies were identified that assessed intervention fidelity, with delivery adherence again being the most frequent concept assessed [20]. In contrast, a review of fidelity in after-school programmes to promote behavioural and academic outcomes identified 55 studies [55]. However, the review of after-school programmes included strategies used to maintain fidelity (e.g. use of an intervention manual) and under further examination, only 29% (*n* = 16) of the included studies actually measured fidelity outcomes. Possible reasons for the lack of attention to fidelity assessment could be a lack of journal space, or a lack of definitive guidance requiring the reporting of fidelity data. The recent development of checklists such as the Template for Intervention Description and Replication (TIDIER) [14] and the Medical Research Council (MRC) guidance on process evaluations [15] may help to improve this situation in the future.

Another key finding from this review was the lack of attention to quality of measures used to assess fidelity in physical activity interventions. Where checklists or rating scales were applied to session recordings or live session observation by researchers, there was a lack of clarity regarding the use of skilled and unbiased raters. Many studies appeared to assume that the use of researchers (who were not necessarily involved in intervention development) was sufficient to evidence rater competence. However, this would not distinguish between junior or experienced researchers and so may introduce a high risk of error. This could also have implications for the validity and reliability of the findings, as it has been shown that (using the MITI coding tool), scoring is more reliable for coders with higher levels of experience than for those with lower levels [56]. There is a further methodological tension here, as those in the best position to rate competence and adherence are arguably the intervention developers themselves or providers (due to the training received in all of the treatment components). However, developers and providers directly involved in the project may have a vested interest in demonstrating high quality of delivery [27] and obtaining skilled independent raters may be more difficult to find for novel interventions. A solution to this dilemma might be to use experts with a wide range of expertise in intervention design or delivery with clear definitions and instructions on the intervention components and their intended use (perhaps with examples of good practice provided by the developers). The use of multiple raters and checking of inter-rater reliability could also help to reduce the risk of bias further.

Only a few studies used objective data collection methods. This may be problematic as objective measures showed poor convergent validity with self-report measures of intervention fidelity in some studies [38, 43]. Factors responsible for this could include social desirability or a lack of sophistication /accuracy /reliability of the measures used. For example, provider self-report measures were typically assessing broad concepts such as a global appreciation of delivery [50], whereas researcher observations assessed more finely-detailed concepts such as the number of specific intervention components delivered [34]. Finally, the lack of attention to the psychometric properties of measurement tools used to assess fidelity also increases the potential for bias due to unknown validity and reliability [57]. This lack of psychometric integrity could be due in part to resource constraints, as novel interventions often require new instruments to be developed. Hence, a balance must be found between scientific rigour and pragmatism.

The importance of assessing intervention fidelity for the interpretation of the findings of intervention effectiveness studies is increasingly recognised [13, 22, 58]. However the current findings suggest that conceptualisation and measurement of intervention fidelity remains heterogeneous in the field of physical activity promotion research [20, 22]. Methodological checklists (e.g. Cochrane) exist to evaluate the quality and rigour of reporting of randomised trials [59]. However, there is currently no such tool for intervention fidelity measurement. Despite this, as demonstrated by this review, the behaviour change consortium [11, 13, 60] have provided a useful basis for categorisation, planning and critical appraisal of intervention fidelity assessments. It is worth noting that studies that used motivational interviewing had access to validated tools with instructions regarding observation method and rater characteristics (e.g. MITI, BECCI). This provides a good example of what might be possible for future development of fidelity assessment methods in the wider field of physical activity interventions. Further research could combine existing tools such as the Behaviour Change taxonomy [61] and the BCC Framework [13] to construct and check the reliability and validity of intervention fidelity checklist items for each behaviour change technique in the intervention. A focus on delivery style as well as content is also important however [62].

### Strengths and limitations

Although previous studies have looked at fidelity to theory [63] and use of fidelity-enhancing strategies [24], to the best of our knowledge this is the first systematic review to specifically identify intervention fidelity assessment methods for physical activity interventions and critique them. Our systematic approach highlighted key conceptual and methodological gaps in current practice. There are however, several limitations of this review that should be acknowledged. First, effectiveness studies may not have reported intervention fidelity in their titles abstracts, or keywords, as implementation is rarely a key focus of intervention studies [31]. This means that it would be possible to miss some relevant studies using the search strategies employed here. However, this review was concerned with understanding how studies typically assessed fidelity and the methodological implications of current practice, rather than aiming to provide a comprehensive overview of the field. It has been previously pointed out, that studies that do not mention fidelity in their titles, abstracts or keywords most likely do not consider it a significant focus [25]. As such, we were confident that the search strategy employed yielded a representative sample of studies in which the issue of fidelity was given significant consideration. Second, although the BCC Framework [13] was used for structuring the analysis, it is worth noting that other conceptual frameworks of intervention fidelity exist, which may have highlighted slightly different issues. For instance, Carrol et al’s (2007) Conceptual Framework for Implementation Fidelity [26] includes content (active ingredients), coverage (reach of the intervention), frequency (number of sessions), duration (time taken), complexity (difficulty), quality of delivery (competence), facilitation strategies (strategies to enhance delivery) and participant responsiveness (participant receipt). Although there is much overlap with the BCC Framework, there are some minor differences. The BCC Framework was used here as it conceptualised intervention fidelity across key stages from design to implementation that are not all covered by Carrol et al’s., (2007) framework. Thirdly, due to the lack of a consensus of reporting standards for fidelity assessment, the level of description of the methods and measures used was generally poor, so ascertaining the provenance and reliability of relevant information was challenging [31]. However, to attempt to mitigate this, a second coder was used to double check the extracted data and companion papers were sourced and included. Finally, the appraisal criteria used were developed for the purposes of this study based on a combination of existing approaches (Table 1), as definitive criteria do not appear to exist in the literature. As such, there may be other important appraisal criteria that were not considered here. However, we hope this will at least provide a building block for further development.

### Implications/recommendations

Based on this review some key recommendations are proposed. Firstly, clearer conceptualisation of fidelity is needed when researchers plan and conduct fidelity analyses. This could be achieved by applying structures such as the BCC Framework. Secondly, researchers should clearly report all aspects of fidelity assessment measures (e.g. observational methods, rater attributes, and sampling procedures) as these can have implications for the likely risk of bias. Thirdly, clearer guidelines are needed on fidelity measurement, including consideration of data collection, sampling, measurement validity and reliability, minimizing the effects of rater bias and other methodological issues. This could then act as an adjunct to existing checklists such as TIDIER. Fourthly, a possible approach for critically appraising fidelity assessment methods in behavioural interventions has been proposed in this review and may provide a useful template for future studies. Finally, researchers should acknowledge the inherent strengths and weaknesses of their assessment methods when reporting and interpreting their intervention fidelity outcomes.

## Conclusion

This review highlights new directions for research to improve the rigour and replicability of behavioural interventions for promoting physical activity by enhancing the assessment of intervention fidelity. The conceptualisation and measurement of fidelity in behavioural interventions for physical activity are wide ranging and of variable quality. Further work is needed to generate a more definitive understanding of the key concepts and best practice methods for conducting fidelity assessments of physical activity (and other behavioural) interventions.

## Additional files

Additional file 1: Search terms with results. Table with search terms for the systematic review with number of results. (DOCX 19 kb)

Additional file 2: Inclusion Criteria. A list of all the relevant inclusion of exclusion criteria. (DOCX 12 kb)

Additional file 3: Data extraction form. Table listing the author, study design, population, outcome, intervention, fidelity measure and fidelity result. (DOCX 82 kb)

## Abbreviations

7-Day PAR: Seven day physical activity recall
BCC: Behaviour Change Consortium
CBT: Cognitive behavioural therapy
CONSORT: Consolidated standards of reporting trials
LTPA: Leisure time physical activity
MI: Motivational interviewing
MITI: Motivational Interviewing Treatment Integrity Scale
MVPA: Moderate to vigorous physical activity
PRISMA: Preferred standards for the reporting of systematic reviews and meta-analyses
TIDIER: Template for intervention description and replication

## References

[1] World Health Organization [WHO]. Physical Activity. 2014; Available from: http://www.who.int/topics/physical_activity/en/

[2] Cooney GM, Dwan K, Greig CA, Lawlor DA, Rimer J, Waugh FR (2013). Exercise for depression. Cochrane Database Syst Rev.

[3] Shaw K, Gennat H, O’Rourke P, Del Mar C. Exercise for overweight or obesity. Cochrane Database Syst Rev. 2006;4 10.1002/14651858.CD003817.pub3 PMC9017288 17054187

[4] Das P, Horton R (2016). Physical activity???time to take it seriously and regularly. Lancet [Internet].

[5] Department of Health. UK physical activity guidlines [Internet]. 2011. Available from: https://www.gov.uk/government/publications/uk-physical-activity-guidelines

[6] Foster C, Hillsdon M, Thorogood M, Kaur A, Wedatilake T. Interventions for promoting physical activity. Cochrane Libr [Internet]. 2009; Available from: http://onlinelibrary.wiley.com/doi/10.1002/14651858.CD003180.pub2/abstract

[7] Greaves CJ, Sheppard KE, Abraham C, Hardeman W, Roden M, Evans PH (2011). Systematic review of reviews of intervention components associated with increased effectiveness in dietary and physical activity interventions. BMC Public Health.

[8] Orrow G, Kinmonth A-L, Sanderson S, Sutton S (2012). Effectiveness of physical activity promotion based in primary care: systematic review and meta-analysis of randomised controlled trials. BMJ.

[9] Mars T, Ellard D, Carnes D, Homer K, Underwood M, Taylor SJC (2013). Fidelity in complex behaviour change interventions: a standardised approach to evaluate intervention integrity. BMJ Open.

[10] Craig P, Dieppe P, Macintyre S, Michie S, Nazareth I, Petticrew M (2008). Developing and evaluating complex interventions: the new Medical Research Council guidance. BMJ.

[11] Bellg AJ, Borrelli B, Resnick B, Hecht J, Minicucci DS, Ory M (2004). Enhancing treatment fidelity in health behavior change studies: best practices and recommendations from the NIH Behavior Change Consortium. Health Psychol.

[12] Moncher FJ, Prinz RJ (1991). Treatment fidelity in outcome studies. Clin Psychol Rev [Internet]..

[13] Borrelli B (2011). The assessment, monitoring, and enhancement of treatment fidelity in public health clinical trials. J Public Health Dent.

[14] Hoffmann TC, Glasziou PP, Boutron I, Milne R, Perera R, Moher D (2014). Better reporting of interventions: template for intervention description and replication (TIDieR) checklist and guide. BMJ.

[15] Moore G, Audrey S, Barker M, Bond L, Bonell C, Cooper C (2014). Process evaluation in complex public health intervention studies: the need for guidance. J Epidemiol Community Health.

[16] Borrelli B, Sepinwall D, Ernst D, Bellg AJ, Czajkowski S, Breger R (2005). A new tool to assess treatment fidelity and evaluation of treatment fidelity across 10 years of health behavior research. J Consult Clin Psychol [Internet].

[17] Schoenwald SK, Garland AF, Chapman JE, Frazier SL, Sheidow AJ (2012). NIH Public Access.

[18] Schoenwald SK, Garland AF (2013). A Review of Treatment Adherence Measurement Methods. Psychol Assess.

[19] Shea OO, Mccormick R, Bradley JM, Neill BO, Shea OO, Mccormick R, et al. Fidelity review: a scoping review of the methods used to evaluate treatment fidelity in behavioural change interventions. Phys Ther Rev. 2016;21(3-6):207-14. Available from: http://www.tandfonline.com/doi/full/10.1080/10833196.2016.1261237.

[20] Schinckus L, Van den Broucke S, Housiaux M (2014). Assessment of implementation fidelity in diabetes self-management education programs: A systematic review. Patient Educ Couns.

[21] Toomey E, Currie-Murphy L, Matthews J, Hurley DA (2015). Implementation fidelity of physiotherapist-delivered group education and exercise interventions to promote self-management in people with osteoarthritis and chronic low back pain: a rapid review part II. Man Ther [Internet].

[22] Gearing RE, El-Bassel N, Ghesquiere A, Baldwin S, Gillies J, Ngeow E (2011). *major* ingredients of fidelity: A review and scientific guide to improving quality of intervention research implementation. Clin Psychol Rev.

[23] Dusenbury L, Brannigan R, Falco M, Hansen WB (2003). A review of research on fidelity of implementation: implications for drug abuse prevention in school settings. Health Educ Res.

[24] Breckon JD, Johnston LH, Hutchison A. Physical activity counseling content and competency: a systematic review. J Phys Act Health. 2008;5(3):(pp 398–417), Available from: https://www.ncbi.nlm.nih.gov/pubmed/18579918. 10.1123/jpah.5.3.398 18579918

[25] Quested E, Ntoumanis N, Thøgersen-Ntoumani C, Hagger MS, Hancox JE (2017). Evaluating quality of implementation in physical activity interventions based on theories of motivation: Current challenges and future directions. Int Rev Sport Exerc Psychol [Internet].

[26] Carroll C, Patterson M, Wood S, Booth A, Rick J, Balain S (2007). A conceptual framework for implementation fidelity. Implement Sci.

[27] Perepletchikova F, Kazdin AE (2005). Treatment integrity and therapeutic change: Issues and research recommendations. Clin Psychol Sci Pract.

[28] Richards J, Hillsdon M, Thorogood M, Foster C (2013). Face-to-face interventions for promoting physical activity. Cochrane Database Syst Rev.

[29] NICE. Behaviour change : individual approaches. 2014;(January):1–14.

[30] Wongpakaran N, Wongpakaran T, Wedding D, Gwet KL (2013). A comparison of Cohen ’ s Kappa and Gwet ’ s AC1 when calculating inter-rater reliability coefficients : a study conducted with personality disorder samples. BMC Med Res Methodol.

[31] Popay J, Roberts H, Sowden A, Petticrew M, Arai L, Rodgers M, et al. Guidance on the conduct of narrative synthesis in systematic reviews. A Prod from ESRC methods Program Version. 2006;1

[32] Aittasalo M, Rinne M, Pasanen M, Kukkonen-Harjula K, Vasankari T. Promoting walking among office employees ― evaluation of a randomized controlled intervention with pedometers and e-mail messages. BMC Public Health. 2012;12(1):403. Available from: BMC Public Health. 10.1186/1471-2458-12-403 PMC3444317 22672576

[33] Albright CL, Saiki K, Steffen AD, Woekel E. What barriers thwart postpartum women’s physical activity goals during a 12-month intervention? A process evaluation of the Nā Mikimiki Project. Women Health. 2015;55(1):1–21. Available from: https://www.ncbi.nlm.nih.gov/pmc/articles/PMC4339404/. 10.1080/03630242.2014.972014 PMC4339404 25402618

[34] Bombardier CH, Ehde DM, Gibbons LE, Wadhwani R, Sullivan MD, Rosenberg DE (2013). Telephone-based physical activity counseling for major depression in people with multiple sclerosis. J Consult Clin Psychol.

[35] Carr LJ, Karvinen K, Peavler M, Smith R, Cangelosi K (2013). Multicomponent intervention to reduce daily sedentary time: a randomised controlled trial. BMJ Open.

[36] Castro C, Pruitt L, Buman M, King A (2011). Physical Activity Program Delivery by Professionals Versus Volunteers: The TEAM Randomized Trial. Heal Psychol [Internet].

[37] Goyder E, Hind D, Breckon J, Dimairo M, Minton J, Everson-Hock E (2014). A randomised controlled trial and cost-effectiveness evaluation of “booster” interventions to sustain increases in physical activity in middle-aged adults in deprived urban neighbourhoods. Health Technol Assess (Rockv).

[38] Hardeman W, Michie S, Fanshawe T, Prevost AT, McLoughlin K, Kinmonth AL (2008). Fidelity of delivery of a physical activity intervention: Predictors and consequences. Psychol Health [Internet].

[39] Kolt GS, Oliver M, Schofield GM, Kerse N, Garrett N, Latham NK. An overview and process evaluation of TeleWalk: a telephone-based counseling intervention to encourage walking in older adults. Health promotion international. 2006;21(3):201-8. Available from: https://academic.oup.com/heapro/article/21/3/201/559161/An-overviewand-process-evaluation-of-TeleWalk-a. 10.1093/heapro/dal015 16702169

[40] Leslie E, Marshall AL, Owen N, Bauman A (2005). Engagement and retention of participants in a physical activity website. Prev Med (Baltim).

[41] Levy SS, Cardinal BJ. Effects of a Self-determination Theory-based Mail-mediated Intervention on Adults’ Exercise Behavior. [Internet]. Baron Fox, Godin, Ingledew, Li, Marcus, Markland, Markland, Ryan, Ryan, Sallis D, editor. Vol. 18, American Journal of Health Promotion. Levy, Susan S.: Department of Exercise and Nutritional Sciences, San Diego State University, 5500 Campanile Dr, San Diego, CA, US, 92182–7251, slevy@mail.sdsu.edu: American Journal of Health Promotion; 2004. p. 345–349. Available from: https://www.ncbi.nlm.nih.gov/pubmed/15163133. 10.4278/0890-1171-18.5.345 15163133

[42] Pinto BM, Goldstein MG, DePue JD, Milan FB (1998). Acceptability and feasibility of physician-based activity counseling. The PAL project. Am J Prev Med.

[43] Quinn L, Trubey R, Gobat N, Dawes H, Edwards RT, Jones C (2016). Development and Delivery of a Physical Activity Intervention for People With Huntington Disease: Facilitating Translation to Clinical Practice. J Neurol Phys Ther [Internet].

[44] Steele RM, Mummery WK, Dwyer T.Examination of program exposure across intervention delivery modes: face-to-face versus internet. International Journal of Behavioral Nutrition and Physical Activity. 2007;4(1):7. Available from: https://www.ncbi.nlm.nih.gov/pubmed/17352817. 10.1186/1479-5868-4-7 PMC1839106 17352817

[45] Williams MA, Williamson EM, Heine PJ, Nichols V, Glover MJ, Dritsaki M, Adams J, Dosanjh S, Underwood M, Rahman A, McConkey C. Strengthening And stretching for Rheumatoid Arthritis of the Hand (SARAH). A randomised controlled trial and economic evaluation. Health Technol Assess. 2015;19(19):1–222. doi: 10.3310/hta19190. 10.3310/hta19190 PMC4780893 25748549

[46] Williams SL, Michie S, Dale J, Stallard N, French DP (2015). The effects of a brief intervention to promote walking on Theory of Planned Behavior constructs: a cluster randomized controlled trial in general practice. Patient Educ Couns [Internet].

[47] Soetens KCM, Vandelanotte C, de Vries H, Mummery KW (2014). Using online computer tailoring to promote physical activity: a randomized trial of text, video, and combined intervention delivery modes. J Health Commun [Internet].

[48] Wilbur J, Schoeny ME, Buchholz SW, Fogg L, Miller AM, Braun LT (2016). Women’s Lifestyle Physical Activity Program for African American Women: Fidelity Plan and Outcomes. J Phys Act Health [Internet]..

[49] Bodde AE, Seo D-C, Frey GC, Lohrmann DK, Van Puymbroeck M (2012). Developing a physical activity education curriculum for adults with intellectual disabilities. Health Promot Pract [Internet].

[50] Brawley LR, Arbour-Nicitopoulos KP, Martin Ginis KA (2013). Developing physical activity interventions for adults with spinal cord injury. Part 3: A pilot feasibility study of an intervention to increase self-managed physical activity. Rehabil Psychol.

[51] Avery L, Charman SJ, Taylor L, Flynn D, Mosely K, Speight J (2016). Systematic development of a theory-informed multifaceted behavioural intervention to increase physical activity of adults with type 2 diabetes in routine primary care: Movement as Medicine for Type 2 Diabetes. Implement Sci.

[52] Bull FC, Milton KE (2010). A process evaluation of a “physical activity pathway” in the primary care setting. BMC Public Health.

[53] McCarthy MM, Dickson VV, Katz SD, Sciacca K, Chyun DA (2015). Process evaluation of an exercise counseling intervention using motivational interviewing. Appl Nurs Res [Internet].

[54] Steele R, Mummery WK, Dwyer T (2007). Using the Internet to promote physical activity: a randomized trial of intervention delivery modes. J Phys Act Health [Internet].

[55] Maynard BR, Peters KE, Vaughn MG, Sarteschi CM (2013). Fidelity in After-School Program Intervention Research: A Systematic Review. Res Soc Work Pract [Internet].

[56] Moyers TB, Martin T, Manuel JK, Hendrickson SML, Miller WR (2005). Assessing competence in the use of motivational interviewing. J Subst Abus Treat.

[57] Terwee CB, Bot SDM, de Boer MR, van der Windt DAWM, Knol DL, Dekker J (2007). Quality criteria were proposed for measurement properties of health status questionnaires. J Clin Epidemiol.

[58] Moore GF, Raisanen L, Moore L, Din NU, Murphy S. Mixed-method process evaluation of the welsh national exercise referral scheme. Health Education. 2013;113(6):476-501. Available from: http://www.emeraldinsight.com/doi/full/10.1108/HE-08-2012-0046.

[59] Higgins JPT, Altman DG, Gotzsche PC, Juni P, Moher D, Oxman a. D, et al. The Cochrane Collaboration’s tool for assessing risk of bias in randomised trials. Bmj. 2011;343(oct18 2):d5928–d5928. 10.1136/bmj.d5928 PMC3196245 22008217

[60] Resnick B, Bellg AJ, Borrelli B, Defrancesco C, Breger R, Hecht J, Sharp DL, Levesque C, Orwig D, Ernst D, Ogedegbe G, Czajkowski S (2005). Examples of Implementation and Evaluation of Treatment Fidelity in the BCC Studies: Where We Are and Where We Need to Go. Ann Behav Med.

[61] Abraham C, Michie S (2008). A taxonomy of behavior change techniques used in interventions. Health Psychol.

[62] Hardcastle SJ, Fortier M, Blake N, Hagger MS. Identifying content-based and relational techniques to change behaviour in motivational interviewing. Health Psychol Rev [Internet]. 2016;7199:1–16. Available from: http://www.tandfonline.com/doi/full/10.1080/17437199.2016.1190659. 10.1080/17437199.2016.1190659 27189713

[63] Keller C, Fleury J, Sidani S, Ainsworth B. Fidelity to theory in PA intervention research. Western Journal of Nursing Research. 2009;31(3):289-311. Available from: https://www.ncbi.nlm.nih.gov/pubmed/19020266. 10.1177/0193945908326067 19020266

[64] Liberati A, Altman DG, Tetzlaff J, Mulrow C, Gøtzsche PC, Ioannidis JPA (2009). The PRISMA statement for reporting systematic reviews and meta-analyses of studies that evaluate health care interventions: explanation and elaboration. J Clin Epidemiol.

[65] Albright CL, Steffen AD, Novotny R, Nigg CR, Wilkens LR, Saiki K (2012). Baseline results from Hawaii’s Nā Mikimiki Project: a physical activity intervention tailored to multiethnic postpartum women. Women Health [Internet].

