# Supplementary material for: Assessment of fidelity in individual level behaviour change interventions promoting physical activity among adults: a systematic review
Source: BMC Public Health. 2017 Oct 2;17:765. doi: 10.1186/s12889-017-4778-6 (PMC5625828; doi:10.1186/s12889-017-4778-6)
Supplement: Supplementary file 1 — Search terms with results. Table with search terms for the systematic review with number of results. (DOCX 19 kb) [file 12889_2017_4778_MOESM1_ESM.docx]

Additional File 1

Search terms with results

Ovid was used to search in PsychINFO, Embase, PsychArticles and MEDLINE

|  | Searches | Results |
| --- | --- | --- |
| 1 | Exercise$.ti,ab,kw. | 586819 |
| 2 | Physical activ$.ti,ab,kw. | 204659 |
| 3 | Swim$.ti,ab,kw. | 75441 |
| 4 | Walk$.ti,ab,kw. | 222239 |
| 5 | Jog$.ti,ab,kw. | 4568 |
| 6 | Run$.ti,ab,kw. | 373908 |
| 7 | sedentary.ti,ab,kw. | 55379 |
| 8 | fidelity.ti,ab,kw. | 44622 |
| 9 | Process evaluation.ti,ab,kw. | 5628 |
| 10 | (Intervention adj3 integrity).ti,ab,kw. | 252 |
| 11 | Treatment adj3 integrity).ti,ab,kw. | 2059 |
| 12 | (Intervention adj3 adherence).ti,ab,kw. | 3718 |
| 13 | (Treatment adj3 adherence).ti,ab,kw. | 28808 |
| 14 | (Intervention adj3 compliance).ti,ab,kw. | 1568 |
| 15 | (Treatment adj3 compliance).ti,ab,kw. | 18421 |
| 16 | (Intervention adj3 implementation).ti,ab,kw. | 5519 |
| 17 | (Treatment adj3 implementation).ti,ab,kw. | 4722 |
| 18 | (Intervention adj3 delivery).ti,ab,kw. | 3522 |
| 19 | (Treatment adj3 delivery).ti,ab,kw. | 13283 |
| 20 | (Intervention adj3 receipt).ti,ab,kw. | 290 |
| 21 | (Treatment adj3 receipt).ti,ab,kw. | 1529 |
| 22 | (Intervention adj3 enactment).ti,ab,kw. | 47 |
| 23 | (Program$ adj3 enactment).ti,ab,kw. | 54 |
| 24 | (Treatment adj3 enactment).ti,ab,kw. | 89 |
| 25 | (Intervention adj3 dose).ti,ab,kw. | 1732 |
| 26 | (Program$ adj3 dose).ti,ab,kw. | 1920 |
| 27 | (Treatment adj3 dose).ti,ab,kw. | 61407 |
| 28 | (Program$ adj3 integrity).ti,ab,kw. | 470 |
| 29 | (Program$ adj3 adherence).ti,ab,kw. | 3264 |
| 30 | (Program$ adj3 compliance).ti,ab,kw. | 3076 |
| 31 | (Program$ adj3 implementation).ti,ab,kw. | 18700 |
| 32 | (Program$ adj3 delivery).ti,ab,kw. | 5306 |
| 33 | (Program$ adj3 receipt).ti,ab,kw. | 123 |
| 34 | 1 or 2 or 3 or 4 or 5 or 6 or 7 | 1325384 |
| 35 | 8 or 9 or 10 or 11 or 12 or 13 or 14 or 15 or 16 or 17 or 18 or 19 or 20 or 21 or 22 or 23 or 24 or 25 or 26 or 27 or 28 or 29 or 30 or 31 or 32 or 33 | 219275 |
| 36 | 34 and 35 | 11464 |
