# Supplementary material for: Assessment of fidelity in individual level behaviour change interventions promoting physical activity among adults: a systematic review
Source: BMC Public Health. 2017 Oct 2;17:765. doi: 10.1186/s12889-017-4778-6 (PMC5625828; doi:10.1186/s12889-017-4778-6)
Supplement: Supplementary file 2 — Inclusion Criteria. A list of all the relevant inclusion of exclusion criteria. (DOCX 12 kb) [file 12889_2017_4778_MOESM2_ESM.docx]

Additional File 2

**Inclusion Criteria**

1. Did the study examine the issue of fidelity (or related terms) of design, training, delivery, receipt or enactment either as a main focus or nested within another study (e.g. trial or feasibility study)?
2. Was the study testing a behavioural intervention designed to increase any type of physical activity at an individual level (i.e. interventions delivered to individuals either singly or in group sessions, but not whole community or whole-population level interventions such as media campaigns or changes in the local environment) as defined as any bodily movement produced by skeletal muscles that requires energy expenditure?
3. Did the intervention focus only on increasing physical activity and no other behaviours (e.g. diet, smoking)?
4. RCTs, observational studies, case-controlled or other quasi-experimental studies. Comparison groups could include usual care, no intervention or other interventions.
5. Was it conducted with adults aged 18 or over;
6. Was it a peer reviewed publication in English?

**Exclude if**

1. Protocol paper, conference abstract, not peer reviewed
2. Intervention also tackled other behaviours (e.g. diet, smoking, relaxation, skills building etc)
3. Intervention was school based or targeted children
4. Study includes physical activity as part of the intervention in addition to the outcome.
5. Study is looking at behavioural support plus physical activity
