# Supplementary material for: Assessment of fidelity in individual level behaviour change interventions promoting physical activity among adults: a systematic review
Source: BMC Public Health. 2017 Oct 2;17:765. doi: 10.1186/s12889-017-4778-6 (PMC5625828; doi:10.1186/s12889-017-4778-6)
Supplement: Supplementary file 3 — Data extraction form. Table listing the author, study design, population, outcome, intervention, fidelity measure and fidelity result. (DOCX 82 kb) [file 12889_2017_4778_MOESM3_ESM.docx]

Additional File 3

| **Author, study design** | **Population, Outcome** | **Intervention** | | **Fidelity Measurement^1^** | | **Fidelity Result^1^** | |
| --- | --- | --- | --- | --- | --- | --- | --- |
| (Aittasalo, Rinne, Pasanen, Kukkonen-Harjula, & Vasankari, 2012),  2 arm RCT | **Population**  241 inactive employees from occupational health care units  **Outcome**  Self-reported weekly minutes of walking. Increase in minutes of “walking for transportation” at 2 months | **Content**  ***Intervention:*** 1 face to face session providing information of health benefits of PA. Pedometers and logbooks given to participants and monthly email message.  ***Control:*** No intervention  **Duration**  6 months  **Delivered by**  Researchers and occupational health care units | **What measured**  ***Delivery:*** Delivery, reading and receiving email messages  ***Enactment:*** Use of pedometers and logbooks  **How measured**  ***Delivery:*** Researcher notes and participant self-report checklists at 2 and 6 months  ***Enactment:*** Researcher notes and participant self-report checklists at 2 and 6 months | | **Fidelity level**  ***Delivery:*** All messages delivered as intended; 80% were read at 6 months.  ***Enactment:*** 60%, 46% of the participants respectively reported having used pedometers and logbooks regularly. 37% and 47% of the participants respectively in reported having used pedometers and logbooks irregularly  **Fidelity Level compared with other outcomes (e.g. physical activity)**  None reported | |  |
| (Albright et al., 2012; Albright, Saiki, Steffen, & Woekel, 2015),  Parallel 2 arm RCT | **Population**  115 inactive healthy postpartum women  **Outcome measure**  MVPA using Active Australia Survey | **Content**  ***Intervention:*** Tailored telephone counselling plus website. Including goal setting, overcoming barriers to MVPA, and the importance of social support.  ***Control:*** Standard website only  **Duration**  17 calls delivered over 12 months  **Delivered by**  Health educator | | **What measured**  ***Delivery:*** Essential intervention components (e.g. goal, setting, social support and problem-solving, goal review). Number of calls made, and time of call. Number of website log ins, number of pages viewed  **How measured**  ***Delivery:*** 5% of calls with fidelity checklist of essential components over 12 months | | **Fidelity Level**  ***Delivery:*** 88% adherence to components. Review pedometer steps asked 68.8%, MVPA resources offered 80% of the time.  Because resources were tailored to the woman's barriers and goals, she may not have required MVPA resources after each call. Discussing barriers to MVPA was covered in 96% of the calls, assessing woman's previous MVPA goal in 97% of calls, and setting the woman's next MVPA goal was discussed in 100% of the evaluated calls.  Compliance to the scheduled telephone calls was high, with 90.4% receiving ≥ 13 of the 17 scheduled calls, with a mean time per call of 12.7 (± 6.4) minutes. Most (78.3%) of the women in TTCW condition viewed the website at least once. They accessed the website 2,092 times for a mean of 17.7 pages viewed per person (among those who viewed website at least once).  **Fidelity Level compared with other outcomes (e.g. physical activity)**  None reported | |
| (Avery et al., 2014, 2016)  1 arm pilot Trial | **Population**  30 patients with type 2 diabetes  **Outcome**  None reported | **Content**  ***Intervention:***  Multifaceted behavioural intervention based on the TBP and SCT. Delivered online, and with discussion cards, booklets, activity planners, DVD’s, Pedometers, Progress pads and leaflets.  **Duration**  4 face to face appointments over 12 months  **Delivered by**  Healthcare professional | | **What measured**  ***Delivery:*** Delivery of the intervention components  **How measured**  ***Delivery:*** Video recordings of 32 consultations were assessed independently by two researchers with expertise in health behaviour change. Checklist assessing presence and absence of specific intervention content (behaviour change techniques). Disagreements were resolved via discussion with the third researcher. Cohen’s kappa calculated for baseline and 1-month coding was 0.60 and 0.55, respectively. | | **Fidelity Level**  ***Delivery:*** Most techniques (i.e. agenda setting, discussion of pros versus cons for increasing PA, and utilisation of importance and confidence rulers) delivered during baseline and 1-month follow-up appointments most of the time. Agenda setting; discussion of pros versus cons for increasing PA; barrier identification and problem-solving; prompt focus on past success; prompt rewards contingent on progress and time management frequently not delivered. Review behavioural goals, delivered during 15 of the 17 1-month follow-up consultations. Prompt generalisation of PA behaviour was not delivered during baseline or 1-month follow-up consultations.  **Fidelity Level compared with other outcomes (e.g. physical activity)**  None reported | |
| Bodde et al., (2012)  1 arm Formative pilot evaluation | **Population**  42 adults with intellectual disabilities  **Outcome**  None reported | **Content**  ***Intervention:*** Multimedia intervention to increase knowledge, skills, and actual control as well as addressing constructs from the TPB. The curriculum included the concept of PA; physical and emotional benefits of PA; PA guidelines; practical ways to increase PA; participating with others; safety; and nutrition.  **Duration**  None reported  **Delivered by**  Instructor | | **What measured**  ***Delivery:*** Script adhered to by provider  ***Receipt****:* Demonstrate the skill and knowledge they acquired.  **How measured**  ***Delivery:*** At four random times throughout the program a research assistant assessed the instructor’s adherence to the script.  ***Receipt****:* Following each lesson process measures used by research team to evaluate each participant’s ability to independently perform the knowledge or skill outlined in the learning objectives. Seven to 10 days later, each participant was evaluated at the beginning of the class for their retention of a previous lesson’s objective. | | **Fidelity Level**  ***Delivery:*** 100% of the material in the script was followed; however, questions and comments from participants interrupted the script as can be expected. Other typical interruptions common to disability day service centres also occurred and included behavioural issues, nonattendance, and emergency drills.  ***Receipt:*** 73.5% to 100% of the participants met the learning objectives in any given session (M = 87.7%). Measurement of selected objectives for retention 7 to 10 days later showed rates of 79.2% to 96.4% (M = 89.8%).  **Fidelity Level compared with other outcomes (e.g. physical activity)**  None reported | |
| (Bombardier et al., 2013)  2 arm RCT | **Population**  92 community-residing adults with multiple sclerosis  **Outcome**  Significantly higher energy expenditure than control using 7 day physical activity recall | **Content**  ***Intervention:*** Face to face and telephone delivered Motivational interviewing intervention.  ***Control:*** Wait list  **Duration**  1 face to face session follow by 7 telephone calls lasting 30 minutes.  **Delivered by**  MI counsellor | | **What measured**  ***Delivery:*** Key indicators of MI fidelity: open questions, closed questions, affirmations, reflections, and summaries. MI-inconsistent behaviours (arguing, confronting, and giving advice without permission). Number of sessions completed and time spent. Subjective ratings were made of the therapist’s MI spirit including warmth, understanding, and egalitarianism.  **How measured**  ***Delivery:*** Random selection of 20% of intervention sessions (n = 65) audio recorded and coded by MI-trained staff using behaviour counts and 7-point scale (from 1, not at all, to 7, very much). | | **Fidelity Level**  ***Delivery:*** Fidelity to MI-consistent behaviours and spirit was good—72% of questions were open rather than closed, and the ratio of reflections to questions was 2.9:1. Both of these indices exceeded standards for MI competency. Mean (SD) frequency of observed therapist behaviours that were MI inconsistent was 0.26 (0.57) per session.  The average number of sessions completed was 6.9 (1.7), and 86.4% of the participants received at least six sessions. The average (SD) time spent in counselling sessions was 138 (59) minutes. Satisfactory for MI spirit (means 5.73–5.88; range 4–7).  **Fidelity Level compared with other outcomes (e.g. physical activity)**  None reported | |
| (Brawley, Arbour-Nicitopoulos, & Martin Ginis, 2013)  1 arm trial | **Population**  13 Adults with spinal cord injury  **Outcome**  Large increase in leisure time PA. Leisure time physical activity questionnaire | **Content**  ***Intervention****:* Face to face group-mediated cognitive–behavioural training intervention based on SCT  **Duration**  9 weeks  **Delivered by**  Interventionist | | **What measured**  ***Delivery****:* Satisfaction with intervention delivery.  ***Receipt****:* Self-regulatory efficacy for scheduling and planning. Understanding of intervention content.  ***Enactment*:** Action plan agreement.  **How measured**  ***Delivery:*** Interventionist rated relative accomplishment of session objective and the participation of group members in each of the eight sessions on a one item, 10-point Likert-type scale. An average per session rating was then calculated.  ***Receipt*:** 11-items measured on a 0 (not at all confident) to 100 (completely confident) percent scale. Mean score was calculated for the scale, with higher scores indicating greater confidence. Internal consistency for the scale was > .80 at both time points. Seven items. Understanding measured on 5 point Likert Scale. Cronbach’s alpha α = .80.  ***Enactment:*** 4–item instrument used to measure the extent to which participants agreed with forming detailed plans to engage in self-managed LTPA in addition to their activity. Each item was rated on a 9-point Likert-type scale. Internal consistency was good (α > .97) at both time points. | | **Fidelity Level**  ***Delivery:*** The interventionist’s ratings of satisfaction for the delivery of the group sessions were high (range = 7 to 10), with a mean rating of 8.38 ± 0.68.  ***Receipt:*** Self-regulatory efficacy changed from a mean of 86.20 (10.49) to 89.43 (10.23) post intervention. Regarding post-intervention perceptions of intervention content, participants strongly agreed that the material presented in the program was usable and easy to understand (M = 4.16, SD = 0.51; 5-point scale).  ***Enactment:*** In terms of their agreement about making action plans for future LTPA, a trend was evident (t (9) = 2.12, p = .06), with an increase about aspects of planning actions for the next four weeks, reflecting a medium- to- large effect (See Table 1). In comparison to baseline, participants more strongly agreed that they had made plans about what, where, when and how they would engage in weekly self-managed LTPA in addition to their supervised, structured LTPA sessions. Relative to the response scale, participants moved from modest disagreement to strong agreement about forming action plans for the upcoming weeks.  **Fidelity Level compared with other outcomes (e.g. physical activity)**  None reported | |
| (Bull & Milton, 2010)  1 Arm Trial | **Population**  378 patients not achieving recommended PA  **Outcome**  General Practice Physical Activity Questionnaire | **Content**  ***Intervention:*** Brief intervention, with adapted MI. Included goal-setting, written resources, and follow-up support.  **Duration**  1 Baseline and 1 follow up consultation  **Delivered by**  General practitioner | | **What measured**  ***Delivery:*** Components of the intervention delivered to each patient. Estimated time taken and **p**ractitioner views and experiences of implementation.  **How measured**  ***Delivery****:* Tracked by data recorded in the Egton Medical Information System (EMIS). EMIS captured data on which. Data were entered by practitioners during or directly after each patient consultation. Focus group discussion undertaken with five practitioners and 5 telephone interviews. Semi-structured interview schedule developed to explore apparent differences between practices in delivery. Focus group and telephone discussions recorded on a digital audio device. | | **Fidelity Level**  ***Delivery:*** Overall each intervention component was provided to the majority of patients, including a discussion on the benefits of physical activity (n = 313), goal setting (n = 295), and signposting to local physical activity opportunities (n = 300). It took on average 20 minutes for patients recruited from the disease registers. These patients received both the screening and BI in the same appointment. Follow-up consultations were estimated to take on average 12 minutes. Practitioner feedback indicated that the delivery of the BI and specifically the use of motivational interviewing varied between practitioners. A lack of confidence and time constraints were cited as the primary barriers to delivering MI consistent consultations.  **Fidelity Level compared with other outcomes (e.g. physical activity)**  None reported | |
| (Carr, Karvinen, Peavler, Smith, & Cangelosi, 2013)  2 Arm RCT | **Population**  40 sedentary overweight adults  **Outcome**  Objective sedentary time measured by StepWatch decreased by 58.7 minutes per day compared to control group | **Content**  ***Intervention:*** Theory based internet delivered programme. Access to a portable pedal machine at their worksite; and a pedometer to use in conjunction with the website.  ***Control:*** Wait list  **Duration**  12 weeks  **Delivered by**  Self-delivered | | **What measured**  ***Delivery*:** Number of website logins  ***Enactment****:* Number of steps logged on the website  **How measured**  Assessed objectively at the end of 12 weeks through a backend tracking database made available by the website administrators. | | **Fidelity Level**  ***Delivery*:** Intervention participants logged on to the website an average of 71.3% (59.8 days) of all days they had access to the website (including weekends; table 4).  **Fidelity Level compared with other outcomes (e.g. physical activity)**  None reported | |
| (Castro, Pruitt, Buman, & King, 2011)  3 Arm RCT | **Population**  181 inactive adults over 50 years of age  **Outcome**  Increase in MVPA at 12 month compared to control measured using the Community Healthy Activities Model Program for Seniors Questionnaire | **Content**  ***Intervention:*** Telephone-based physical activity advice delivered by a trained professional staff member. Guided self-management program delivered via one face-to-face session followed by scheduled telephone contacts. Grounded in SCT and TTM.  *Intervention:* Identical advice delivered by a trained volunteer peer mentor  ***Control:*** Attention control  **Duration**  12 months  **Delivered by**  Professional staff and peer mentors | | **What measured**  ***Delivery*:** Intervention content delivered in each session. Participant perceived quality and competence of providers. Intervention quantity (length of session).  **How measured**  ***Delivery*:** Providers completed structured contact sheets to quantify the intervention content delivered in each session. Intervention quality included (self-management concepts discussed, tip sheets sent to participant; goals set) of the intervention delivered. Ten content areas were identified included perceived benefits of physical activity; problem-solving barriers to physical activity; discussion of lessons learned from previous physical activity experiences; “pros” versus “cons” of being active; injury prevention; enhancing enjoyment of physical activity; self-rewards for being active; enhancing self-efficacy through overcoming obstacles and building success; eliciting social support; and implementing relapse prevention. The intervention director verified the intervention fidelity data through weekly review of audio taped sessions and contact sheets. Participants rated perceived quality and competence of providers across both intervention arms using a 39-item scale adapted from previous studies. The scale was comprised of items that assessed participants’ perceived trust, competence, communication, empowerment, and connection to their advisor. Cronbach’s coefficient alpha at baseline was .98, indicating high internal consistency of items. | | **Fidelity Level**  ***Delivery*:** Relative to professional staff, peer mentors more frequently discussed by phone the balance of pros and cons of physical activity; the perceived benefits of PA, physical activity history and self-rewards. Professional staff more frequently discussed self-efficacy in the phone contacts relative to the peer mentors.  In rating their perceptions of their advisor’s skill and competency, there were. No significant between group differences or changes over time across the two arms of participants perceived quality and competence of providers. Peer mentors and staff delivered equal amounts of the intervention, completing an average of 11 of 14 planned telephone calls across the year. Average length of the telephone calls was equivalent between arms, averaging approximately 15–16 minutes.  **Fidelity Level compared with other outcomes (e.g. physical activity)**  None reported | |
| (Goyder et al., 2014)  3 arm, parrallel-group, pragmatic, RCT | **Population**  282 previously sedentary people aged 40–64 years, living in deprived areas of Shefﬁeld, UK  **Outcome**  No significant between group differences in TEE | **Content**  *Intervention:* A ‘full booster’ group receiving two face-to-face physical activity consultations, provided in a MI style, underpinned by self-determination theory.  ***Control:*** A control group who received no intervention after randomisation.  **Duration**  2 months  **Delivered by**  None reported | | **What measured**  ***Delivery:*** Counts of MI adherent and non-adherent behaviours. Global ratings of evocation, collaboration, autonomy/support, direction and empathy.  **How measured**  ***Delivery:*** 4 interventionists assessed for after training and at 9 and 18 months using the MITI. Sessions were independently coded by a qualiﬁed MITI coder. | | **Fidelity Level**  ***Delivery:*** The reﬂection to question ratio increased across the four interventionists who completed delivery of the intervention from phase 1 to phase 2. The use of directional and deeper complex reﬂections was rated moderate or below competence across all interventionists. Mostly characterised as proﬁcient for direction and competent for other global MI measures. Technical aspects of MI, including the use of open questions, increased across all interventionists from baseline. The global rating of ‘direction’ was consistently high across all interventionists at phase 1 and phase 2.  **Fidelity Level compared with other outcomes (e.g. physical activity)**  MI ﬁdelity was associated with physical activity as measured by mean TEE per day in kcal at 3 months (p = 0.027). | |
| (Hardeman et al., 2008; Kinmonth et al., 2008)  3 Arm RCT | **Population**  365 sedentary adults with a parental history of Type 2 Diabetes  **Outcome**  Energy expenditure measured by heart rate. Increased in both intervention groups at an equivalent of 20 minutes of brisk walking a day. | **Content**  ***Intervention:*** Delivered face to face Participants taught to maximise personal advantages and opportunities, and to minimise disadvantages and obstacles to becoming more physically active. The intervention focused on eight self-regulatory strategies for behavioural change, including goal-setting, action-planning, self-monitoring, using rewards, goal-review, using prompts, building support from family and friends, and prevention of relapses.  ***Intervention*:** Same as above but over telephone  ***Control:*** Advice only  **Duration**  12 months  **Delivered by**  Facilitator | | **What measured**  ***Delivery:*** All behaviours specified in the protocol coded under 12 behaviour change techniques and two communication techniques. Delivery of nine techniques (Building motivation, goal setting, action planning, self-monitoring, rewards, goal review, prompts, relapse prevention and habit formation)  ***Receipt:*** Confidence in using each strategy  ***Enactment:*** Use of eight self-regulatory strategies  **How Measured**  ***Delivery:*** Validated checklist expressed as the number of component behaviours within a technique applied by the facilitator, divided by the number of behaviours specified by the protocol. 52 participants selected purposively from both intervention arms. Final sample of 27 participants. Independent blinded rater assessed 108 transcripts and a second rater assessed sessions one and four for all participants. Inter-rater agreement on behaviours in sessions one and four was over 75% for 88% (76/86) of behaviours (range: 63–100%). Median agreement on techniques was 86% (IQR: 79–92%). Levels of agreement were similar for session one and four and intra-class correlation was high at 0.96 for all behaviours across sessions. There was no drift in assessment by the independent rater, with inter-rater agreement over 75% for 16 transcripts randomly selected for review by the first author. Facilitators recorded delivery of each technique (‘covered’ or ‘not covered’) after each session.  ***Receipt****:* Measured with 10 items (α = 0.93) on a scale from 1 (not at all confident) to10 (very confident). Items were summed to calculate overall scores.  ***Enactment:*** Questionnaires at baseline and 6 months (93% response) assessed the use of eight self-regulatory strategies (yes/no): Goal setting, action planning, self-monitoring, using rewards, goal review and using prompts (one item each) and building support and relapse prevention (two items each) (0.77 for all 10 items combined). | | **Fidelity Level**  ***Delivery:*** Mean adherence to techniques across all sessions ranged from 25% (generalising skills to other behaviours, e.g., healthy eating) to 66% (summarising, defining the agenda), with an overall mean of 45%. General communication skills were used most often, followed by techniques to facilitate initial behaviour change techniques to facilitate maintenance and generalising skills to other behaviours, e.g., healthy eating, were used least frequently, both within and across sessions. Use of techniques by facilitators varied considerably across participants (18–71%), with a median (IQR) of 44% (35–62%).  100% for facilitator-self reported adherence (IQR 97–100%).  ***Enactment:*** Most participants in the combined intervention groups reported at both 6 and 12 months that they had used the eight behaviour change strategies. More than 75% reported that they had set goals and used action plans and self-monitoring activities, and more than 60% reported they had used family support and techniques for dealing with setbacks.  **Fidelity Level compared with other outcomes (e.g. physical activity)**  No significant associations between observed facilitator adherence to individual techniques and all techniques combined, and participants’ cognitions about increasing physical activity and confidence in using self-regulatory strategies at 6 months or change in these variables between baseline and 6 months. No significant correlations fond for observed adherence and change in intention, affective attitude, and levels of objective and self-reported PA. | |
| (Kolt et al., 2006)  2 Arm RCT | **Population**  186 sedentary older adults (mean age 74).  **Outcome**  Auckland Heart Study Physical Activity Questionnaire. | **Content**  *Intervention:* Telephone counselling and print based intervention based on the TTM, CBT and MI.  *Control:* No treatment.  **Duration**  3 months.  **Delivered by**  Motivational counsellor. | | **What measured**  ***Delivery:*** Participant perception of counsellor support**.**  **How measured**  ***Delivery:*** 63 intervention group participants completed and survey focused on counsellor support, which was assessed in terms of counsellor advice being helpful and/or relevant, and whether the counsellor provided a service that was motivating, understanding, supportive and/or professional. | | **Fidelity Level**  ***Delivery:*** All respondents agreed or strongly agreed that the counsellor was understanding and supportive, and that a good overall level of service and support was provided. 97% agreed or strongly agreed that the service was professional, and 95% reported that the advice provided was helpful and the counsellor was motivating in terms of becoming and remaining physically active.  **Fidelity Level compared with other outcomes (e.g. physical activity)**  None reported | |
| (Leslie, Marshall, Owen, & Bauman, 2005)  (Marshall, Leslie, Bauman, Marcus, & Owen, 2003)  2 Arm RT | **Population**  655 University academic and general staff.  **Outcome**  No significant change in IPAQ scores between or within groups. | **Content**  ***Intervention:*** Website and email based intervention based on the TTM.  ***Intervention:*** Print delivery of same intervention.  **Duration**  10 weeks.  **Delivered by**  Self-delivered. | | **What measured**  ***Delivery:*** How many website visits, how long spent on website, how many pages viewed, how many emails received and read and how many modules of the website read.  **How measured**  ***Delivery:*** Website usage statistics. Specific items given to participants who recalled receiving any e-mails about the project were asked to recall how many e-mails they received, how much of them they read. For the website, participants were asked to rate how many sections of the website they read how often they accessed the website. | | **Fidelity Level**  *Delivery*: 152 participants visited the website at least once, the average time spent browsing the website was 9 min and the average number of pages viewed was 18. 227 received at least one e-mail and 23% of these recalled seeing all four e-mails. 115 of the follow-up sample recalled the website and 27% recalled there were four sections to the website.  **Fidelity Level compared with other outcomes (e.g. physical activity)**  None reported | |
| (Levy & Cardinal, 2004)  3 Arm RCT | **Population**  185 adults not achieving PA recommendations.  **Outcome**  Increased PA for women indicated by the Leisure time exercise questionnaire. | **Content**  ***Intervention:*** Mailed information packet based on SDT. Behavioural and cognitive strategies promoting a sense of autonomy, competence, and relatedness.  ***Intervention:*** Additional booster card mailed.  ***Control:*** Informational packet containing American Heart Association (AHA) physical activity and health facts adapted from the AHA website.  **Duration**  2 months  **Delivered by**  Self-delivered | | **What measured**  ***Delivery:*** Received and read information packet.  ***Enactment:*** Completion of worksheets  **How measured**  ***Delivery:*** Participants reported whether they had received the packet (and, if appropriate, booster postcard) that was mailed to them, whether they had read the packet or postcard.  ***Enactment:*** Participants reported whether they had completed intervention packet worksheets (in intervention groups only). | | **Fidelity Level**  ***Delivery:*** 83.7%of those providing feedback reported receiving and reading their respective packets, with no differences observed across conditions.  ***Enactment:*** 35.3% and 57.9% of those in the intervention or intervention-plus-booster conditions reported completing the self-help worksheets included with their print materials.  **Fidelity Level compared with other outcomes (e.g. physical activity)**  None reported | |
| (McCarthy, Dickson, Katz, Sciacca, & Chyun, 2015)  Pre=post pilot study | **Population**  20 adults with heart failure**.**  **Outcome**  None reported | **Content**  ***Intervention:*** Brief face-to-face exercise counselling followed by weekly telephone follow up based on MI, and the use of a daily diary for self-monitoring. Participants were given an accelerometer to keep track of step-counts, 2-pound hand weights with instructions for upper body exercises, and a diary to record the four self-care activities.  **Duration**  12 weeks.  **Delivered by**  MI counsellor. | | **What measured**  ***Delivery:*** Fidelity to the intervention and use of MI. Quantity or amount of intervention delivered to participants.  ***Enactment*:** Use of the daily diary.  **How measured**  ***Delivery***: All 20 exercise counselling sessions conducted at the beginning of the study were audiotaped. Four audiotapes were sent to an independent expert in MI for review and assessment of the interventionist’s use of MI. Each tape was scored for adherence to the principles of MI. Summary scores for each of the four audiotapes included ﬁve categories: (1) average of spirit global (use of evocation, collaboration, autonomy/support, direction, and empathy); (2) reﬂection to question ratio; (3) percent open questions; (4) percent complex reﬂections; and (5) percent MI-adherent. Evaluation of the dose of the intervention that was delivered consisted of examining the quantity or amount of intervention delivered to participants.  ***Enactment:*** Engagement with the daily dairy was tallied for each of four activities: daily step counts, body weight, use of the hand weights, and the Borg scale. The total number of actual recorded data for each activity was divided by the number of potential diary recordings. | | ***Delivery:*** Scoring of the tapes from subject 3 and 4 that were sent at revealed an overall low adherence to MI principles (50% and 40%). Percentage of open questions, was 40% and 25%. Reﬂections-to-questions ratio less than 1:1 (considered beginning MI proﬁcient). Global spirit rating was poor. The initial session, lasted approximately one hour. The mean time was 12.57 minutes. 168 calls (93%) were made successfully and step-count data were collected. The length of each call lasted approximately 5 minutes. The mean number of calls over 12 weeks for each subject was 16.3.  ***Enactment:*** Participants recorded step-counts 64% of days and body weight 52% of days. The Borg scale was recorded 50% of days and the use of hand weights was recorded 32% of days. These data reﬂect compliance with the diary recordings, not necessarily what was actually done for that day.  **Fidelity Level compared with other outcomes (e.g. physical activity)**  None reported | |
| (Pinto, Goldstein, DePue, & Milan, 1998)  2 Arm RCT | **Population**  **355** Older adults  **Outcome**  None reported | **Content**  ***Intervention:*** Activity counselling delivered by community based on a patient-centred model and the stages of change approach  ***Control:*** No intervention  **Duration**  1 session plus follow up  **Delivered by**  Primary care physicians | | **What measured**  ***Training:*** Confidence in providing activity counselling  ***Delivery*:** Extent to which they provided speciﬁc components of activity counselling to all their patients  **How measured**  ***Training:*** Prior to randomization, physicians were asked to complete a brief questionnaire assessing their conﬁdence in providing activity counselling. A summary score was computed as a mean of the eight items’ ratings.  ***Delivery:*** Physicians were administered a post-intervention questionnaire after completing the follow-up visits, which included a self-reported evaluation of activity counselling. At follow-up interviews, patients were asked a series of questions about the activity counselling that they may have received. | | **Fidelity Level**  ***Training:*** There was a signiﬁcant difference between groups in summary score change over time with the IG physicians showing increased conﬁdence in providing exercise counselling.  ***Delivery:*** Most physicians reported counselling 75% of their patients across all counselling behaviours. Ninety-three percent of patients who provided data at 6 weeks reported receiving activity counselling from their physician during the initial visit. Patients reported that the physician spent an average of 8.9 minutes counselling them about exercise.  **Fidelity Level compared with other outcomes (e.g. physical activity)**  None reported | |
| (Busse et al., 2014; Quinn et al., 2016)  2 Arm RCT | **Population:** 46 participants with genetically confirmed Huntington disease  **Outcome**  None reported | **Content:**  ***Intervention:*** Programme face to face based on SDT comprising of a purpose developed workbook, and an exercise DVD.  ***Control:*** Social contact control intervention  **Duration**  14 weeks  **Delivered by**  Coaches | | **What measured**  ***Delivery:*** Whether the content of each of the sessions was consistent with what was speciﬁed in the protocol and number of minutes**.** Extent to which each coach demonstrated efforts to promote a patient’s autonomy, relatedness, and competence.  **How measured**  ***Delivery:*** Self-report checklist after each home visit. Full audio recording of one of the coach home visits and audio-recordings one of their later home visits third of 6 visits). Rated independently rated by a member of the study team, using a 0 to 4 rating scale for the 3 SDT areas and overall impression of the coach’s performance. Used to provide coaches with constructive feedback on their interactions and as a mediating factor in analysis. To ensure that the ﬁdelity rating tool could be readily implemented in a clinical setting utilizing relatively novice raters, the study team member (who was a researcher and not involved in delivery of the intervention) and the intervention coordinator independently rated 3 audio ﬁles and compared ratings for agreement. The ratings for the 2 raters were within one point of each other, and for 2 of the 3 total scores, there was 100% agreement. Fidelity of the intervention was further evaluated by asking coaches to complete a self-assessment of their perceived ability to deliver the intervention as intended. | | **Fidelity Level**  ***Delivery:*** In 100% of sessions coaches introduced participants to the workbook, gave the participants the exercise DVD and discussed the concept of goal-setting. Sessions lasted on average 72.3 minutes.  Overall scores ranged from 7 to 14 out of a possible 16 points, with a mean (standard deviation) score across the coaches of 11.0 (2.4). Coach interactions scored an average of 2.5 for autonomy, 3.0 for relatedness, 2.7 for competence, and 2.8 for the overall impression. Self-assessment scores were 3.1 for autonomy, 3.3 for relatedness, and 3.0 for competence.  **Fidelity Level compared with other outcomes (e.g. physical activity)**  Self-assessment scores were on average higher than those assigned by the independent rater. | |
| (Soetens, Vandelanotte, de Vries, & Mummery, 2014)  3 Arm RT | **Population**  803 Australian adults  **Outcome**  Physical activity measured by the Active Australia Survey improved significantly over time in all groups | **Content**  ***Intervention:*** Internet delivered video intervention based on the TPB and TTM  ***Intervention:*** Internet delivered text intervention based on the TPB and TTM  ***Intervention*:** Internet delivered text and video intervention based on the TPB and TTM  **Duration**  1 month  **Delivery by**  Self-delivered | | **What measured**  ***Delivery:*** Time spent on the website for each participant during the entire intervention period  **How measured**  ***Delivery:*** Website user statistics | | **Fidelity Level**  ***Delivery:*** Participants in the video group spent significantly more time in total on the website during the intervention period (21.4 minutes) than participants in the text group (13.4 minutes) and the combination group (15.1 minutes).  **Fidelity Level compared with other outcomes (e.g. physical activity)**  None reported | |
| (Steele & Mummery, 2007)  3 Arm RT | **Population**  192 inactive adults  **Outcome**  Physical activity measured by the Active Australia Survey | **Content**  ***Intervention:*** Health-eSteps intervention based on SCT and included techniques such as; lifestyle activity, benefits and barriers, goal setting, self-monitoring, resistance training, self-talk, self-reinforcement, time and stress management, relapse prevention, and social support.  ***Intervention:*** FACE group received weekly 1-hour face-to-face contact sessions with a trained program  ***Intervention:*** Face to face plus internet. Received the same content delivered via the internet, and two additional face-to-face sessions.  **Duration**  10 weeks  **Delivery by**  Facilitator | | **What measured**  ***Delivery:*** Number of face to face sessions and logins to the website  **How measured**  ***Delivery:*** Web tracking was used to track exposure across both intervention groups. | | **Fidelity Level**  ***Delivery:*** The mean number of face-to-face sessions (exposure) attended by the FACE group was 6.1 The mean number of logins (exposure) for the IM group was 11.5 and 11.8 for the IO group.  **Fidelity Level compared with other outcomes (e.g. physical activity)**  None reported | |
| (JoElIen Wilbur et al., 2016; Joellen Wilbur et al., 2016)  3 Arm RCT | **Population**  288 African American Women  **Outcome**  Physical activity measured by the Community Health Activities Model Programme for Seniors (CHAMPS) and using accelerometers. Main effect for increase in physical activity | **Content**  ***Intervention (overall):*** Six 2-hour group meetings. Based on SCT and MI. An easy-to-read participant manual for home use supplemented the group content.  ***Group 1*:** Above, with Personal motivational calls  ***Group 2*:** Above with Automated messages  ***Group 3*:** No calls between meetings  **Duration**  48 weeks  **Delivered by**  Delivered predominately by registered nurses who were research staff members. | | **What measured**  ***Delivery:*** Checklist of whether or not the interventionist performed the expected action outlined in the study manual (adherence). Competence (e.g. actively engages women in all discussion)  ***Enactment:*** Assessing participants’ self-monitoring of their lifestyle PA prescription (steps they walked) using accelerometer.  **How measured**  ***Delivery:*** Each group meeting recorded with a digital audio recorder by the interventionist and given to clinical psychologist. Sample of group meetings and a combination of observation and digital audio recordings. The goals were to (1) get a reasonable estimate of intervention fidelity; (2) provide feedback to the interventionists based on initial evaluations; and (3) avoid longitudinal effects such as drift. Fidelity was assessed for 3 of the 6 group meetings for each of the 18 cohorts. Adherence: 12 to 16 checklist items. Summed and divided by the total number of items for percent adherence. Above 75% was considered adherent. Selected checklist items included the following: discussed benefits and trade-offs of physical activity, discussed activities women. Competence: 14 scored from 1 to 3. Items summed and divided by 14, with a possible range of 1 to 3. A score above 2.5 was competent. In a community based parenting intervention, the measure showed high interrater agreement for both adherence and competence scales (94% and 85%, respectively) and adequate intraclass correlation coefficients (adherence = 0.69, competence = 0.91).The average of these 3 assessments formed the fidelity score for each cohort.  PC implementation fidelity delivery was assessed with the MI Treatment Integrity Code Version 3.0 (MITI). A global rating score and behaviour counts.  Interventionists and research assistants entered each participant’s attendance at group meetings held in the community into a web based tracking system. Likewise, the PC interventionists logged their MI calls into a web-based tracking system. The system was programmed to provide reports on attendance at group meetings and completion of PCs by participant, study condition, and cohort. This allowed assessment of intervention receipt or dose. The outcaller of the ATCL was programmed to deliver ACs related to motivational messages during the adoption and maintenance phase of the intervention for the AC condition. All calls were stamped with the date and outcome: (1) voice (connected to a voice) and motivational message selected, (2) busy, (3) no answer, (4) no ring back, (5) no dial tone, (6) answering machine, or (7) hung-up. The incaller of the ATCL was also programmed to stamp the date of all incoming calls that connected to a motivational message. This information was downloaded and stored as Excel data so that the number of PCs from the incaller and outcaller that connected to a motivational message could be counted.  ***Enactment:*** Data were exported from software at the group meeting for analysis. Thus, it was possible to identify how many weeks the women actually wore their accelerometers. | | **Fidelity Level**  ***Delivery:*** % of adherence to the group meeting protocol across all 3 conditions averaged greater than 70%, with no differences by condition. Interventionists’ competence scores at the group meeting for all 3 conditions were close to the maximum score of 3, with no differences across conditions. Examination of MI revealed that the global clinician rating score was close to 4, suggesting competence in delivering MI. The overall percentage of open-ended questions, was a mean of 46% of all questions, and complex reflection was just 2% of all reflections. The ratio of reflections to questions tended to be low (less than 1). A mean of 81% of behaviors were MI-adherent, which is below the threshold for beginning proficiency (90%).  ***Enactment:*** 71.9% of the women wore their accelerometer more than 24 weeks out of a total of 48 weeks.  **Fidelity level compared with other outcomes (e.g. physical activity)**  Adherence in delivering the group meeting showed no significant effects on change in PA. A ceiling effect in the competence of group leaders made it infeasible to assess the impact on outcomes. The global clinician rating for fidelity delivery of PCs showed no significant change in PA. | |
| (Williams, Michie, Dale, Stallard, & French, 2015)  2 Arm Cluster RCT | **Population**  315 general practice patients  **Outcome**  No significant differences in walking measured by pedometer | **Content**  ***Intervention:*** Face to face based on self-regulation intervention plus information pack.  ***Control:*** Patients received the information pack detailed above, and were also offered the opportunity to discuss their own walking with the practice nurse.  **Duration**  6 weeks  **Delivery by**  Practice nurse | | **What measured**  ***Training:*** Provider competence before trial  **How measured**  ***Training:*** Assessed by research team using a 20-point checklist of the intervention techniques. Practice nurses and HCAs were required to achieve a minimum level of competence of delivery, which was that 12/14 intervention components were delivered correctly. | | **Fidelity Level**  None reported  **Fidelity Level compared with other outcomes (e.g. physical activity)**  None reported | |

^1^Fidelity Measures and Results are organised according to the categories of the Borrelli framework (Bellg et al., 2004; Borrelli, 2011; Borrelli et al., 2005) with any “other” non-framework categories noted separately.
